# Does Oxidative Stress Along with Dysbiosis Participate in the Pathogenesis of Asthma in the Obese?

**DOI:** 10.1007/s12013-022-01114-z

**Published:** 2022-11-08

**Authors:** Paulina Kleniewska, Rafał Pawliczak

**Affiliations:** Department of Immunopathology, Faculty of Medicine, Medical University of Lodz, Zeligowskiego 7/9, 90-752 Lodz, Poland

**Keywords:** Reactive oxygen species, Oxidative stress, Biomarkers

## Abstract

The most important environmental factor that can play a key role in the development of asthma in the obese is overproduction of reactive oxygen species (ROS). The aim of the study was to examine changes in the concentration of oxidative stress parameters in the lungs, bronchoalveolar lavage (BAL) fluid and blood of mice in models of asthma or/and obesity caused by high-fat diet (HFD). The concentrations of 4-HNE and isoprostanes in the lungs of the animals were measured. BAL fluid levels of hydrogen peroxide were marked. Additionally, thiobarbituric acid reactive substances (TBARS) and ferric reducing ability of plasma (FRAP) were used as biomarkers of oxidative stress in the blood. Administration of lipoic acid (LA), a probiotic with standard-fat diet (SFD, 10% fat) and low-fat diet (LFD, 5% fat) significantly decreased the concentration of 4-HNE as compared to the OVA (ovalbumin) + HFD group (*p* < 0.05). Treatment with low-fat diet or LFD in combination with apocynin insignificantly decreased H_2_O_2_ values as compared to the OVA + HFD group. Supplementation of probiotic with SFD and LFD significantly decreased the concentration of TBARS as compared to the OVA + SFD and saline + HDF groups (*p* < 0.05). Significantly lower concentrations of TBARS were also observed in the LA plus LFD group (*p* < 0.05) as compared to the OVA + HFD group. Low-fat diet with probiotic significantly increased the concentration of FRAP as compared to the obese mice (*p* = 0.017). Treatment with LFD in combination with LA significantly increased FRAP values as compared to the obese and obese asthmatic mice (*p* < 0.001).

## Introduction

The impact of reactive oxygen species (ROS) on cellular components depends on the concentration and duration of action. In higher concentrations, they are involved in the processes leading to the damage of the main cellular elements, disorders of the structure and function of the cell, and ultimately its death [1, 2]. Therefore, presence of free radicals (FRs) is related to the body’s defense mechanisms and their useful activity. However, an excess of free radicals is definitely harmful since exceeding certain ROS concentrations in cells leads to development of many diseases. Radicals react quickly with various molecules often causing their damage. A particularly threatening factor for cells is oxidative stress (OS) [3, 4]. It occurs as a result of imbalance between the production of ROS and their removal by the body’s antioxidant mechanisms or repair of an existing damage. Several studies have shown that OS occurs in the course of many diseases, including asthma and obesity [5, 6].

Both asthma and obesity are diseases associated with chronic inflammation and overproduction of ROS. Therefore, this combination may lead to increased OS resulting in the propagation of airway inflammation through redox sensitive sites in the nuclear transcriptional pathways, favoring the Th2-mediated cytokine response and promoting epigenetic changes that impair the activity of histone deacetylases. Obesity can affect lung function, the immune system and mediators involved in airway function, leading to development of asthma in obese individuals. Obesity changes the mechanics of the lungs. This is due to the aggravating effect of visceral adipose tissue on the diaphragm and of adipose tissue on the chest wall. Importantly, airway reactivity can be triggered by breathing with a small lung volume. Obesity may increase airway oxidative stress through obesity-mediated adipokine imbalance associated with higher levels of leptin and lower levels of adiponectin. It is accompanied by intensification of systemic OS, also occurring in the respiratory tract.

NADPH oxidase is an enzyme involved in the production of ROS in the respiratory system. The superoxide anion (O_2_^−.^) generated by this enzyme is not only one of the most reactive oxygen species, but it also has the ability to transform into H_2_O_2_, and by reacting with NO, it contributes to the formation of peroxynitrite (ONOO^−^). The best known inhibitor of this protein is apocynin (acetovanillone; APO). This compound blocks the p47phox oxidase subunit and is one of the most important H_2_O_2_ “scavengers”. APO is characteristic for its selective action that does not interfere with the body’s natural defense. It has been proved that this inhibitor reduces the production of O_2_^−.^ from activated neutrophils and macrophages. APO inhibits the formation of ONOO¯ which is responsible for epithelial damage and the release of inflammatory mediators [7]. Chiang et al. [8] and Hsu et al. [9] reported that APO attenuates lung injury by downregulating ROS generation and the downstream signaling pathways. Stefanska et al. [10] showed that following administration of APO, H_2_O_2_ concentration significantly decreased in exhaled breath condensate in asthmatics.

Antioxidants, such as lipoic acid-LA, prevent the initiation of the oxidative process, inhibit initiated free radical reactions, remove oxidative damage and protect the cell against toxic ROS. They maintain pro- and anti-oxidant balance in the course of many diseases in which free radicals are produced in excessive amounts.

A number of recently published papers discuss the relationship between an intestinal dysbiosis and the development of bronchial asthma or obesity [11]. It has also been proved that the dysbiosis occurring in obese people may be avoided by low-energy diet [12, 13]. Some data may suggest that modulation of the gut microbiota might provide a novel treatment target in obesity coexisting with asthma. The supplementation of some prebiotics, probiotics or synbiotics may have an impact on the composition of gut microbiota and the regulation of gene expression in the muscle, liver and adipose tissues. The aim of the study was to assess some of the oxidative stress parameters in the lungs, BAL fluid and blood of obese asthmatic mice and to investigate the effect of probiotic/antioxidant supplementation combined with an appropriate diet on this process.

## Materials and Methods

### Chemicals

OVA-albumin from chicken egg white (>98%, grade V), (±)-a-lipoic acid synthetic (>99%), acetovanillone, ferric chloride, 2,3,5-triphenyltetrazolium chloride, acetate buffer (pH = 3,6), hydrochloric acid, thiobarbituric acid, trichloroacetic acid, butylated hydroxytoluene, butyl alcohol, sulfuric acid and other chemical reagents were purchased from Sigma-Aldrich-Poland. A lipid peroxidation (4-HNE) Assay Kit (Item No. ab238538) and a Hydrogen Peroxide Assay Kit (Item No. ab102500) were obtained from ABCAM. An 8-Isoprostane ELISA Kit (Item No. 516351) was obtained from Cayman Chemical Company, Ann Arbor, MI.

### Experimental Procedure

As discussed in a previous study [14], animals (adult, male C57/BL6 mice) were randomly divided into ten groups (*n* = 7/group). In group 1, the mice received standard-fat diet (SFD) with saline, group 2 received SFD with ovalbumin—OVA, group 3 received high-fat diet (HFD) with saline, group 4 received HFD with OVA, group 5 received HFD with OVA and apocynin per os, group 6 received HFD with OVA and then low-fat diet (LFD), group 7 received HFD with OVA and apocynin per os with LFD, group 8 received HFD with OVA and probiotic per os with LFD, group 9 received HFD with OVA and lipoic acid per os with LFD, group 10 received HFD with OVA and probiotic per os with standard-fat diet (SFD). All the therapeutic interventions lasted 12 weeks.

All the experimental procedures were approved by the Ethics Committee of the Medical University of Lodz (No. 26/ŁB59/2017).

### Biochemical Determinations

#### Measurement of 4-HNE concentration in the pulmonary tissues

To measure 4-HNE concentration in the pulmonary tissues, a lipid peroxidation (4-HNE) Assay Kit (Item No. ab238538), manufactured by ABCAM (Symbios Sp. z o.o., 83-010 Straszyn, ul. Modrzewiowa 37, Poland), was used (https://www.abcam.com/lipid-peroxidation-4-hne-assay-kit-ab238538.html). The lipid peroxidation (4-HNE) Assay Kit consisted of the following components: 4-HNE-BSA Standard, Anti-4-HNE Antibody (1000X), Secondary Antibody, HRP Conjugate (1000X), Assay Diluent, 10X Wash Buffer, Substrate and Stop Solution, 4-HNE Conjugate, 100X Conjugate Diluent and Protein Binding Strip Well Plate. At first, 50 µl of Standard or Sample to wells of 4-HNE Conjugate coated plate were added. The plate was incubated for 10 min. Then 50 µl of the diluted anti-4-HNE antibody was added to the wells. The plate was incubated for 1 h. In the next stage, the plate was washed with 250 µl 1X Wash Buffer. Then, 100 µl of diluted Secondary Antibody-HRP Conjugate per well was added, followed by 1-h incubation. The washing procedure was repeated. Next, 100 µl of warm Substrate Solution was added. The cuvette was incubated for 20 min. Finally, 100 µl of Stop Solution was added to each well. Absorbance was read at 450 nm immediately with a plate reader.

#### Measurement of 8-isoprostanes concentration in the pulmonary tissues

To measure 8-isoprostanes concentration in the pulmonary tissues, an 8-Isoprostane ELISA Kit (Item No. 516351) manufactured by Cayman Chemical Company, Ann Arbor, MI (BIOKOM, ul. Wspolna 3, 05-090 Janki, Poland), was used (https://www.caymanchem.com/product/516351/8-isoprostane-elisa-kit). The 8-Isoprostane ELISA Kit consisted of the following components: 8-isoprostane ELISA Standard, 8-isoprostane-AchE Tracer, 8-isoprostane Antiserum, ELISA Buffer Concentrate, Wash Buffer Concentrate, Polysorbate 20, Mouse anti-rabbit IgG coated plate, ELISA Tracer Dye, ELISA Antiserum Dye and Ellman’s reagent (5,5′-dithiobis-(2-nitrobenzoic acid), DTNB). A suggested plate format with some modifications (NSB—non- specific binding; B0—maximum binding; Blk- blank; S_1_–S_8_—standards; TA—total activity; number—samples) was used. At first, 100 µl of ELISA Buffer was added to NSB wells and 50 µl of this buffer was added to B0 wells. Next, 50 µl of ELISA Standard was added to 1–8 (S_1_–S_8_) wells and 50 µl of sample was added to wells. Then, 50 µl of AChE Tracer was added to wells, except for Blk and TA wells. Also, 50 µl of 8-isoprostane ELISA Antiserum was added to each well, except for NSB, Blk and TA wells. The plate was incubated for 18 h at 4 °C. The next step was to wash the plate five times with Wash Buffer. Then, 200 µl of DTNB was added to each well and 5 µl of tracer was added only to the TA wells. The assay developed in 120 min in the dark at room temperature using an orbital shaker. The absorbance was measured at the wavelength of 420 nm.

#### Measurement of H_2_O_2_ concentration in the bronchoalveolar lavage

To measure H_2_O_2_ concentration in the bronchoalveolar lavage fluid, the Hydrogen Peroxide Assay Kit (Item No. ab102500) manufactured by ABCAM (Symbios Sp. z o.o., 83-010 Straszyn, ul. Modrzewiowa 37, Poland), was used (www.abcam.com/ab102500). The Hydrogen Peroxide Assay Kit consisted of the following components: H_2_O_2_ Standard, H_2_O_2_ Assay Buffer, OxiRed Probe (in DMSO) and HRP. For each reaction, 50 µl of Reaction Mix was prepared. Sufficient reagents were mixed for the number of tests to be performed (samples, standards and background control). The reaction mix consisted of 46 μl of Assay Buffer, 2 μl of OxiRed Probe and 2 μl of HRP. In total, 50 µl of the Reaction Mix was added into each well. The cuvette (protected from light) was incubated for ten min. Absorbance was read at 570 nm with a plate reader.

#### Measurement of ferric reducing ability of plasma (FRAP)

To determine the FRAP values, the original method described by Benzie and Strain [15] was used, with some modifications. A working FRAP reagent was prepared by mixing 10 ml of acetate buffer (300 mM/pH 3.6) with 1 ml of TPTZ (10 mM dissolved in 40 mM HCl) and 1 ml of FeCl_3_ (20 mM). Following the incubation (37 °C), 30 µl of the mice plasma and 90 µl of deionized water were added to 900 µl of the FRAP reagent. Absorbance was measured at 593 nm with a spectrophotometer (Perkin-Elmer Lambda 25) and monitored for 10 min.

#### Measurement of thiobarbituric acid reactive substances (TBARS) in the mice serum

To measure TBARS, a mixture containing 1000 µl of H_2_SO_4_ (0.05 M), 500 µl of TCA (1.23 M) and 100 µl of serum were prepared. The whole content was mixed and then centrifuged 1500 rpm for 10 min at 4 °C. Subsequently, the supernatant was discarded and the residue was mixed with 2000 µl of distilled water, 10 µl of butylated hydroxytoulene (0.01%) and 500 µl of TBA solution. At the next stage, the obtained mixture was incubated for 30 min in a water bath at 100 °C. After cooling, 2500 µl of 1-butanol was added to the tubes and the samples were shaken intensely. Then they were centrifuged at 1500 rpm for 10 min at 20 °C. Measurements were made in the supernatant of the butanol layer. Fluorescence was measured at an emission wavelength of 546 nm and an excitation wavelength of 515 nm.

### Statistical Analysis

Differences between the groups were assessed using one-way ANOVA followed by Dunnett’s method, Tukey’s multiple-range test or Duncan’s method as a post hoc tests. Dunn’s method was performed following rejection of the Kruskal-Wallis test. All the data were presented as the mean ± standard deviation (SD); *p* value below 0.05 was considered significant.

## Results

### Evaluation of 4-HNE Values

**Fig. 1:**
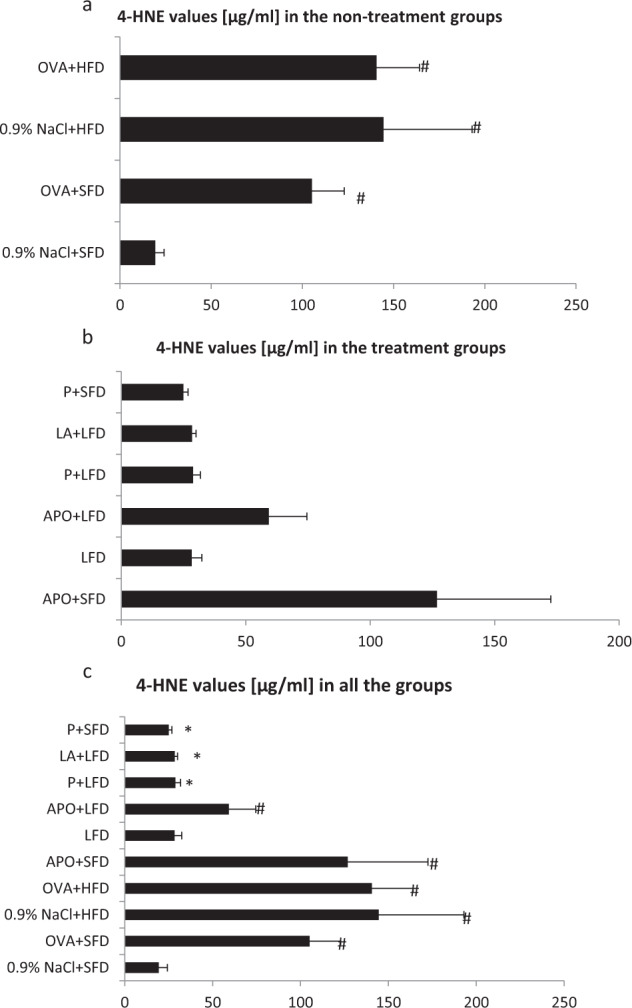
4-HNE levels in all the groups of animals. Data are shown as mean ± SD. **a** 4-HNE values [µg/ml] in the non-treatment groups (respectively: standard-fat diet group—control, standard-fat diet group with ovalbumin, high-fat diet group and high-fat diet group with ovalbumin); **b** 4-HNE values in the treatment groups (respectively: administration of apocynin—15 mg/kg with standard-fat diet; supplementation of low-fat diet alone; administration of apocynin—15 mg/kg with low-fat diet; supplementation of probiotic with low-fat diet; administration of lipoic acid—100 mg/kg with low-fat diet; supplementation of probiotic with standard-fat diet); **c** 4-HNE values [µg/ml] in all the groups of animals; ^#^*p* < 0.05 vs. the 0.9% NaCl + SFD group; *p < 0.05 vs. the OVA + HFD group (Dunn’s method)

The 0.9% NaCl + HFD group, OVA + SFD group and the OVA + HFD group demonstrated significantly higher 4-HNE values than the controls (*p* < 0.05). Administration of a probiotic with standard-fat diet (10% fat) and low-fat diet (5% fat) significantly decreased the concentration of 4-HNE as compared to the OVA + HFD group (*p* < 0.05). Remarkably lower concentrations of 4-HNE were also observed in the lipoic acid plus low-fat diet group (*p* < 0.05) as compared to the OVA + HFD group (Fig. 1).

### Evaluation of 8-isoprostane Values

**Fig. 2:**
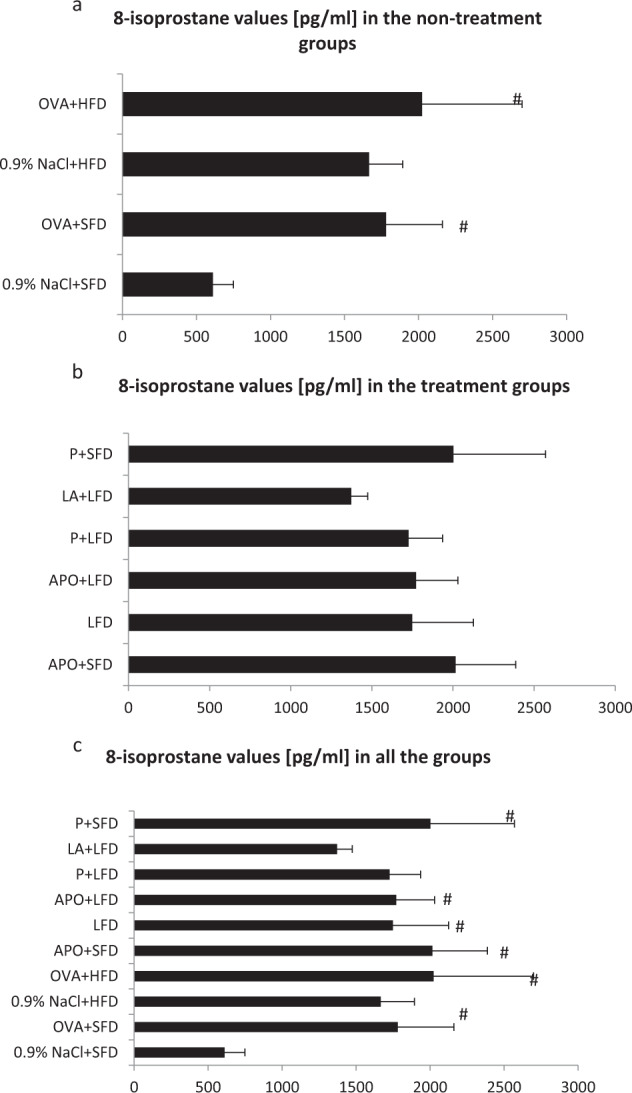
8-isoprostane levels in all the groups of animals. Data are shown as mean ± SD. **a** 8-isoprostane values [pg/ml] in the non-treatment groups (respectively: standard-fat diet group—control, standard-fat diet group with ovalbumin, high-fat diet group and high-fat diet group with ovalbumin); **b** 8-isoprostane values in the treatment groups (respectively: administration of apocynin—15 mg/kg with standard-fat diet; supplementation of low-fat diet alone; administration of apocynin—15 mg/kg with low-fat diet; supplementation of probiotic with low-fat diet; administration of lipoic acid—100 mg/kg with low-fat diet; supplementation of probiotic with standard-fat diet); **c** 8-isoprostane values [pg/ml] in all the groups of animals; ^#^*p* < 0.05 vs. the 0.9% NaCl + SFD group (Dunn’s method)

In the OVA + SFD and OVA + HFD groups the levels of 8-isoprostane were elevated significantly above the control values (*p* < 0.05). The 0.9% NaCl + HFD group also demonstrated an insignificantly higher isoprostane value than that found in the controls. Figure 2 shows that the mice treated with low-fat diet with lipoic acid demonstrated lower levels of 8-isoprostane than the OVA + HFD group. However, no significant changes were observed.

### Evaluation of Hydrogen Peroxide Values

**Fig. 3:**
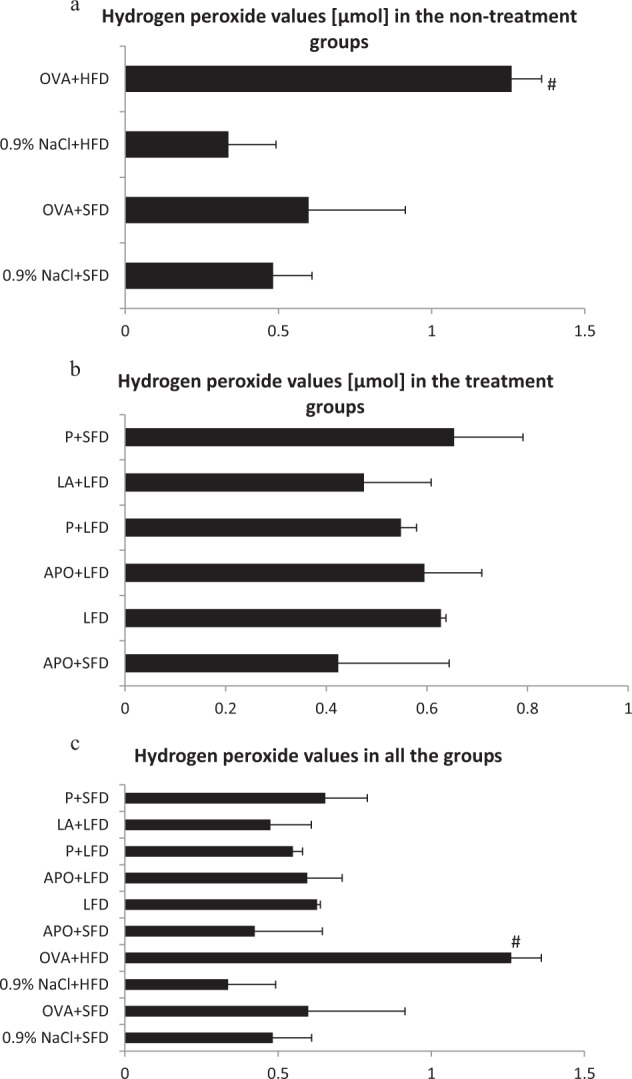
H_2_O_2_ levels in all the groups of animals. Data are shown as mean ± SD. **a** H_2_O_2_ values [µmol] in the non-treatment groups (respectively: standard-fat diet group—control, standard-fat diet group with ovalbumin, high-fat diet group and high-fat diet group with ovalbumin); **b** H_2_O_2_ values in the treatment groups (respectively: administration of apocynin—15 mg/kg with standard-fat diet; supplementation of a low-fat diet alone; administration of apocynin—15 mg/kg with low-fat diet; supplementation of probiotic with low-fat-diet; administration of lipoic acid—100 mg/kg with low-fat diet; supplementation of probiotic with standard-fat diet); **c** H_2_O_2_ values [µmol] in all the groups of animals; ^#^*p* < 0.05 vs. the 0.9% NaCl + SFD group (Dunn’s method)

Levels of H_2_O_2_ in the BAL fluid of the obese asthmatic animals were significantly higher than those of the control group (*p* < 0.05). Figure 3 shows that the mice treated with low-fat diet or LFD plus LA or LFD plus probiotic demonstrated lower levels of BAL hydrogen peroxide than the OVA + HFD group. However, no significant changes were observed. Treatment with low-fat diet or LFD in combination of apocynin insignificantly decreased H_2_O_2_ values as compared to the OVA + HFD group (*p* > 0.05).

### Evaluation of TBARS Values

In the 0.9% NaCl + HFD group, the levels of TBARS were elevated significantly above the control values (*p* = 0.012). The OVA + SFD group and the OVA + HFD group also demonstrated insignificantly higher TBARS values than the controls.

**Fig. 4:**
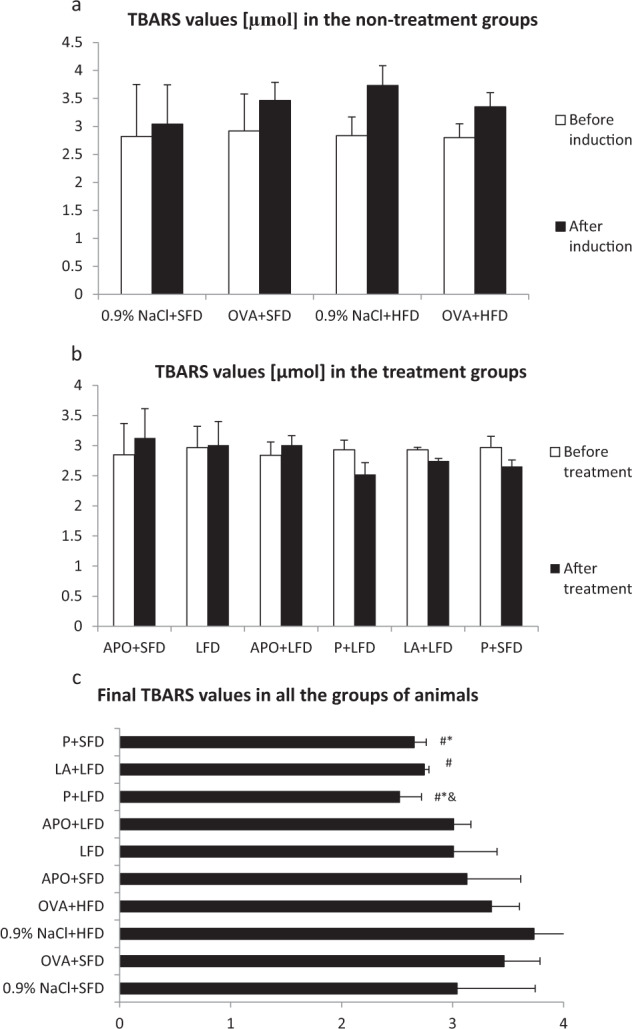
TBARS levels in all the groups of animals. Data are shown as mean ± SD. **a** TBARS values [µmol] in the non-treatment groups (respectively: standard-fat diet group—control, standard-fat diet group with ovalbumin, high-fat diet group and high-fat diet group with ovalbumin); **b** TBARS values [µmol] in the treatment groups (respectively: administration of apocynin—15 mg/kg with standard-fat diet; supplementation of a low-fat diet alone; administration of apocynin—15 mg/kg with low-fat diet; supplementation of probiotic with low-fat diet; administration of lipoic acid—100 mg/kg with low-fat-diet; supplementation of probiotic with standard-fat-diet); **c** Final TBARS values [µmol] in all the groups of animals; ^#^*p* < 0.05; vs. the 0.9% NaCl + HFD group; *p < 0.05 vs. the OVA + SFD group; ^&^*p* < 0.05 vs. the OVA + HFD group (Dunn’s method)

Administration of a probiotic with SFD and LFD significantly decreased the concentration of TBARS as compared to the OVA + SFD and 0.9%NaCl + HDF groups (*p* < 0.05). Significantly lower concentrations of TBARS were also observed in the lipoic acid plus low-fat diet group (*p* < 0.05) as compared to the OVA + HFD group. The administration of low-fat diet, either alone or in combination with apocynin, resulted in a decrease in TBARS concentration as compared to the 0.9% NaCl+HFD and OVA + HFD groups. However, these changes were not significant (Fig. 4).

### Evaluation of FRAP Values

Levels of FRAP in the plasma of the obese and obese asthmatic animals were significantly lower than those of the control group (*p* = 0.013; *p* = 0.042, respectively).

**Fig. 5:**
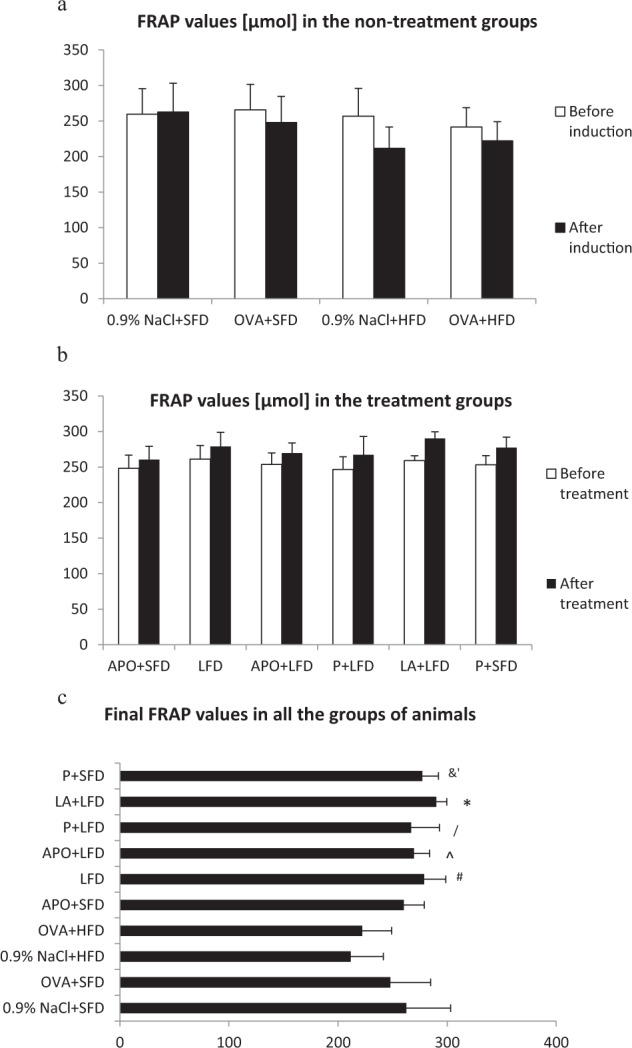
FRAP levels in all the groups of animals. Data are shown as mean ± SD. **a** FRAP values [µmol] in the non-treatment groups (respectively: standard-fat diet group—control, standard-fat diet group with ovalbumin, high-fat diet group and high-fat diet group with ovalbumin); **b** FRAP values [µmol] in the treatment groups (respectively: administration of apocynin—15 mg/kg with standard-fat diet; supplementation of a low-fat diet alone; administration of apocynin—15 mg/kg with low-fat diet; supplementation of probiotic with low-fat diet; administration of lipoic acid—100 mg/kg with low-fat diet; supplementation of probiotic with standard-fat-diet); **c** Final FRAP values [µmol] in all the groups of animals; *p < 0.01; ‘*p* = 0.006; /*p* = 0.017 vs. the 0.9% NaCl + HFD and OVA + HFD groups; ^#^*p* = 0.005; ^&^*p* = 0.018; ^^^*p* = 0.033 vs. OVA + HFD (Tukey test)

Figure 5 shows that the mice treated with LFD demonstrated higher levels of plasma FRAP than the OVA + HFD group (*p* = 0.005). Treatment with low-fat diet in combination of apocynin significantly increased FRAP values as compared to the 0.9% NaCl + HFD group (*p* = 0.011) and OVA + HFD group (*p* = 0.033).

Supplementation of low-fat diet with probiotic significantly increased the concentration of FRAP as compared to the obese mice (*p* = 0.017). The level of FRAP increased also in the probiotics plus standard-fat diet group vs. the OVA + HFD group (*p* = 0.018) and the 0.9 % NaCl + HFD group (*p* = 0.006).

Treatment with low-fat diet in combination with lipoic acid significantly increased FRAP values as compared to the obese and obese asthmatic mice (*p* < 0.001).

## Discussion

This study shows that the values of 4-HNE, isoprostanes and hydrogen peroxide were significantly higher in the high-fat diet group with OVA than in the standard-fat diet controls. The correlation between oxidative stress biomarkers and obesity was investigated by Araki et al. [16]. Plasma concentration of isoprostanes was measured in two groups of Japanese children and adolescents. The obtained results proved that 8-isoprostane concentration was significantly higher in the obese group than in the controls (*p* < 0.001). Kim et al. [17] have proved that asthmatic airways are characterized by an elevated level of 4-HNE.

Our present findings indicate that the values of TBARS were significantly higher in the high-fat diet group than in the standard-fat diet controls. An insignificant increase in TBARS level was also observed in the OVA + SFD and OVA + HFD groups as compared to the controls. Also, the levels of FRAP in the plasma of the obese and obese asthmatic animals were significantly lower than those of the control group. These findings are consistent with recent studies. Elmarakby and Imig [18] confirm that plasma TBARS values were elevated in high-fat diet-fed rats as compared to controls. Kolyva et al. [19] have proved that obesity increased the plasma TBARS and protein carbonyls levels in septic patients. Hajiluian et al. [20] have found that HFD significantly induced oxidative stress by increasing MDA concentrations and reducing glutathione peroxidase, superoxide dismutase activity in the rat hippocampus. Moreover, Delwing-de Lima et al. [21] have observed that HFD enhanced TBARS and protein carbonyl content in the blood, however, moderate-intensity continuous training and high-intensity interval training prevented an increase in TBARS levels. Another study also proves that physical training may reduce the concentration of malondialdehyde [22].

Additionally, Nadeem et al. [23] confirmed that plasma lipid peroxides levels were increased in acute exacerbations of asthma. Other studies also report that asthmatic patients show increased lipid peroxidation products indicating higher oxidative stress [24]. Liu et al. [25] have reported that air pollution may increase airway oxidative stress parameters such as TBARS and decrease small airway function in asthmatic children.

Our data show that treatment with lipoic acid in combination with low-fat diet significantly increased FRAP values as compared to the obese and obese asthmatic mice. Moreover, significantly lower concentrations of TBARS were observed in the group administered lipoic acid plus low-fat diet as compared to the OVA + HFD group. Cho et al. [26] confirm that LA may be useful as an adjuvant therapy for bronchial asthma. BALB/c mice treated with LA had a significantly reduced AHR and a lower proportion of eosinophils among BAL cells. The compound also relevantly decreased concentrations of IL-4 and IL-5 in BAL, serum OVA-specific IgE concentrations, intracellular ROS levels and nuclear factor kappaB DNA-binding activity.

In vitro studies show that LA is a good scavenger of singlet oxygen, hypochlorous acid, peroxynitrite and hydroxyl radicals. Its metabolites, such as bisnorliponate and tetranorliponate, also remove FRs [27]. This antioxidant increases the therapeutic properties of other drugs such as APO - apocynin [28]. The combination of this compound with selenium also brings beneficial effects in gentamicin-induced neurotoxicity [29]. The substance acts as an anti-inflammatory agent, it affects the activity of the NF-kB transcription factor which leads to a decrease of the rate at which inflammatory response mediators are produced [30]. Some authors [31] have shown that LA inhibits IkB kinase degradation and expression of NF- kB dependent genes, regardless of the antioxidant properties of this compound. Golbidi et al. [32] suggest that LA has an inhibitory effect on NF-kB activation induced by both tumor necrosis factor and forbol esters.

The mentioned compound stimulates receptors activated by a-peroxisome proliferators and y-peroxisomes [33, 34]; they have been shown to be involved in the proliferation and differentiation of many cells, as well as in the course of inflammation. Moreover, this acid reduces histamine secretion from mast cells as a result of calcium absorption inhibition and increased cAMP levels [35]. Some researchers [36] note that LA’s anti-inflammatory effect is associated with its antioxidant activity in various tissues. New studies demonstrate significant effects of LA in endotoxemia [37–39].

The beneficial properties of LA related to prevention and/or treatment of obesity are associated, among others, with the inhibition of cAMP-activated protein kinase in the hypothalamus. Reducing the activity of this enzyme in the brain leads to an increase in energy expenditure and reduction of food absorption [40, 41]. A similar impact of LA on cAMP-activated protein kinase activity was observed in skeletal muscles [42], however, not in the liver. Butler et al. [33] report that this antioxidant increases lipase activity in the liver and reduces glycerolipid content, thus limiting the secretion of very low-density lipoproteins rich in triglycerides. Moreover, the compound increases insulin-stimulated glucose uptake, triggers anaerobic glucose conversion in adipocytes and reduces plasma leptin concentration in obese animals [43]. All these activities reduce food intake and increase the body’s overall energy expenditure. The authors have reported that this antioxidant causes weight loss in obese people by about 2% [44].

Our data show that the administration of probiotics with standard-fat diet and with low-fat diet significantly decreased the concentration of TBARS as compared to the OVA + SFD and 0.9%NaCl + HDF groups. Moreover, the supplementation of low-fat diet with probiotics significantly increased the concentration of FRAP as compared to obese mice. The level of FRAP increased also in the probiotics plus standard-fat diet group vs. the OVA + HFD group and the 0.9% NaCl + HFD group.

Recently, published papers indicate a relationship between the microbiome and the asthma symptoms occurring. Most of intestinal bacteria are strains that have a positive effect on the body, however, sometimes they play a different role. Presence of specific bacterial strains can adversely affect the host. This “adverse effect” is most often caused by changes in the composition of all microorganisms occurring in a given habitat or/and is associated with a decrease in the number of commensal bacteria. Many authors describe that factors exerting a direct impact on the above condition include, among others, reactive oxygen species overproduction (oxidative stress), comorbidities, improper diet or environmental conditions (pollution).

Dysbiosis as a phenomenon of the non-physiological composition of the microflora may be the cause of the development of allergies, obesity or bronchial asthma [45, 46]. Scientific data indicate significant differences between the composition of the intestinal microflora of healthy infants and those who suffer from asthma [46]. Less than a decade ago, it was proved that early exposure to environmental microorganisms protects against the development of asthma [47]. It has been shown that one of many factors significantly lowering the risk of asthma is exposure to selected microorganisms during infancy. Other factors showing a beneficial effect include having older siblings [48], having domestic animals [49], growing up in close contact with farm animals [47, 50], consumption of unpasteurized milk [51]. A lower incidence of asthma has also been reported in breast-fed children [52, 53]. Bacteria such as Staphylococcus, Bacteroides, Clostridium and Enterobacteriaceae are associated with an increased risk of atopy [54].

In conclusion, various studies conducted in recent years suggest a relationship between oxidative stress, intestinal dysbiosis and development of bronchial asthma in obese people. The performed experiments not only extend the knowledge about the causes and development of the disease but also indicate the potential options of its treatment and prevention. Supplementation with probiotic bacterial strains, such as Lactobacillus, and antioxidants, such as LA, may be very useful in developing new methods supporting the treatment of bronchial asthma (extended by modulation of intestinal microflora).

## Abbreviations

4-HNE: 4-hydroxynonenal
APO: apocynin
BAL: bronchoalveolar lavage
FRAP: ferric reducing ability of plasma
FRs: free radicals
HFD: high-fat diet
ISO: isoprostanes
LA: lipoic acid
LFD: low-fat diet
OS: oxidative stress
OVA: ovalbumin
P: probiotic
ROS: reactive oxygen species
SFD: standard-fat diet
TBARS: thiobarbituric acid reactive substances
